# Effect of visual feedback during laparoscopic basic training using a box trainer with a transparent top

**DOI:** 10.1002/ags3.12010

**Published:** 2017-06-07

**Authors:** Kosei Maemura, Yuko Mataki, Hiroshi Kurahara, Yota Kawasaki, Shinichirou Mori, Satoshi Iino, Masahiko Sakoda, Shinichi Ueno, Hiroyuki Shinchi, Shoji Natsugoe

**Affiliations:** ^1^ Department of Digestive Surgery, Breast and Thyroid Surgery Field of Oncology Kagoshima University Graduate School of Medical and Dental Sciences Kagoshima Japan; ^2^ Clinical Oncology Kagoshima University Graduate School of Medical and Dental Sciences Kagoshima Japan; ^3^ Kagoshima University Graduate School of Health Sciences Kagoshima Japan

**Keywords:** laparoscopic surgery, simulation training, visual feedback

## Abstract

Laparoscopic surgical training using a box trainer facilitates mastery of laparoscopic surgery. Few studies have investigated whether visualizing the surgical field in the box trainer improves performance of laparoscopic surgical procedures during laparoscopic training. An original box trainer equipped with a transparent top made of mesh covered with a latticed structure was developed and used for evaluation of novices during laparoscopic training. Three tasks (levels 1 to 3) involving organ handling while setting the surgical field were arranged to evaluate the efficacy of training. Forty‐five students were divided into three groups: group A, students without practical training; group B, students trained using the covered box trainer; and group C, students trained using the transparent box trainer. Completion time of each task before and after training was compared. Training significantly reduced the operating time, with a significant difference between the level 1 task and the levels 2 (*P*<.001) and 3 (*P*<.0001) tasks. There was no significant difference in operating time between the levels 2 and 3 tasks. Overall time reduction rate in group C was significantly shorter than that in group A, but not in group B. The time reduction rate for the level 3 task was lowest in group C, with a statistically significant difference existing in group A (*P*<.001). Visual feedback during surgery through the transparent top of the laparoscopic box trainer helped reduce the learning time required to carry out laparoscopic surgery.

## 1 INTRODUCTION

Laparoscopic surgery is one of the fastest growing areas in surgery and has become an important choice for most surgical treatment.^1^, ^2^, ^3^, ^4^ Surgical training to acquire technical skills and ensure patient safety is well known to be beneficial in nonclinical settings.^5^, ^6^, ^7^, ^8^ However, naive operators have some difficulties in learning to carry out and manage laparoscopic surgical procedures. Novice surgeons need to overcome the lack of stereopsis, restricted haptic feedback, difficult handling of unfamiliar instruments with the fulcrum effect, and looking over the working space.^5^, ^9^

The training simulator for laparoscopic surgery is generally classified into two categories: the classical box trainer and the computer simulator. The box trainer is made of common materials such as plastic and/or steel and is equipped with an external, charged‐coupled device camera and a display monitor.^10^, ^11^ The simple structure is low cost and easy to manipulate, despite the variation in type of simulator used.^12^, ^13^ Trainees experience the haptic feedback as real as in live surgery through physical handling. The computer simulator has a virtual reality system that simulates various patterns of surgical training programs with or without haptic feedback.^14^, ^15^ According to several reports, the box trainer has equal effectiveness for fundamental laparoscopic training as the computer simulator, despite the cost difference between the two devices.^16^, ^17^, ^18^

The visual surgical condition is quite different between the laparoscopic and open approach. An important reason for the necessity of laparoscopic training is to become familiar with the visual limitation. However, only a few studies have investigated whether visualizing the surgical field through the transparent top of the box trainer helps to familiarize the trainer with laparoscopic surgical procedures.^19^ The primary goal of the present study was to verify whether laparoscopic training with real‐time visual feedback through a transparent view is useful to master laparoscopic surgical organ handling for novices.

## 2 MATERIALS AND METHODS

### 2.1 The original box trainer

**Figure 1:**
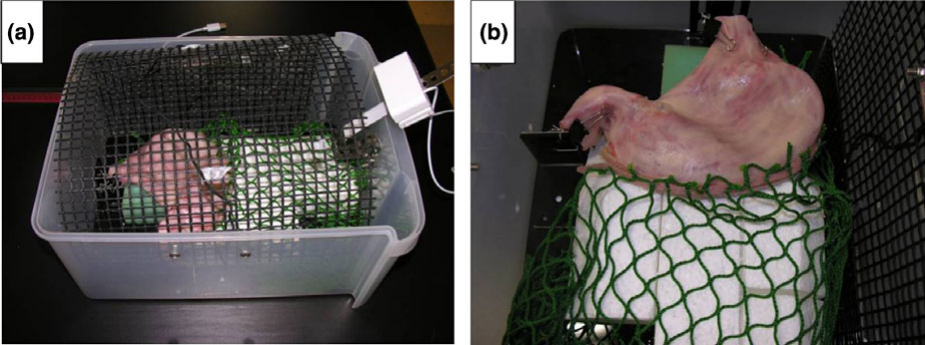
External appearance and internal structure of the original laparoscopic box trainer. (A) Canopy of the box, which is made of mesh with a latticed structure and equipped with a USB camera, and (B) simulated organ unit mounted in the box trainer as the simulated intra‐abdominal cavity

To train novice surgeons, we developed the original box trainer to simulate organ handling during laparoscopic surgery (patent publication number 2011‐113056). The size of the box is 40 cm (length) × 30 cm (width) × 20 cm (height), as shown in Figure 1A. As the canopy of the box is made of mesh that is covered with a latticed structure measuring 8 mm on each side, the inside of the box could be viewed through the top. The inside of the box was visualized by using a USB camera (UCAM‐A1D30MNSV; Elecom Co., Ltd, Osaka, Japan) connected to the notebook computer. The simulated organ unit was made with a silicon stomach equipped with a removable chemical fiber net that replicated the omentum. This organ model was mounted in the box trainer to simulate the intra‐abdominal cavity (Figure 1B). The oral side of the simulated stomach was fixed to the base of the box to simulate the transection at the duodenum.

### 2.2 Training tasks

**Figure 2:**
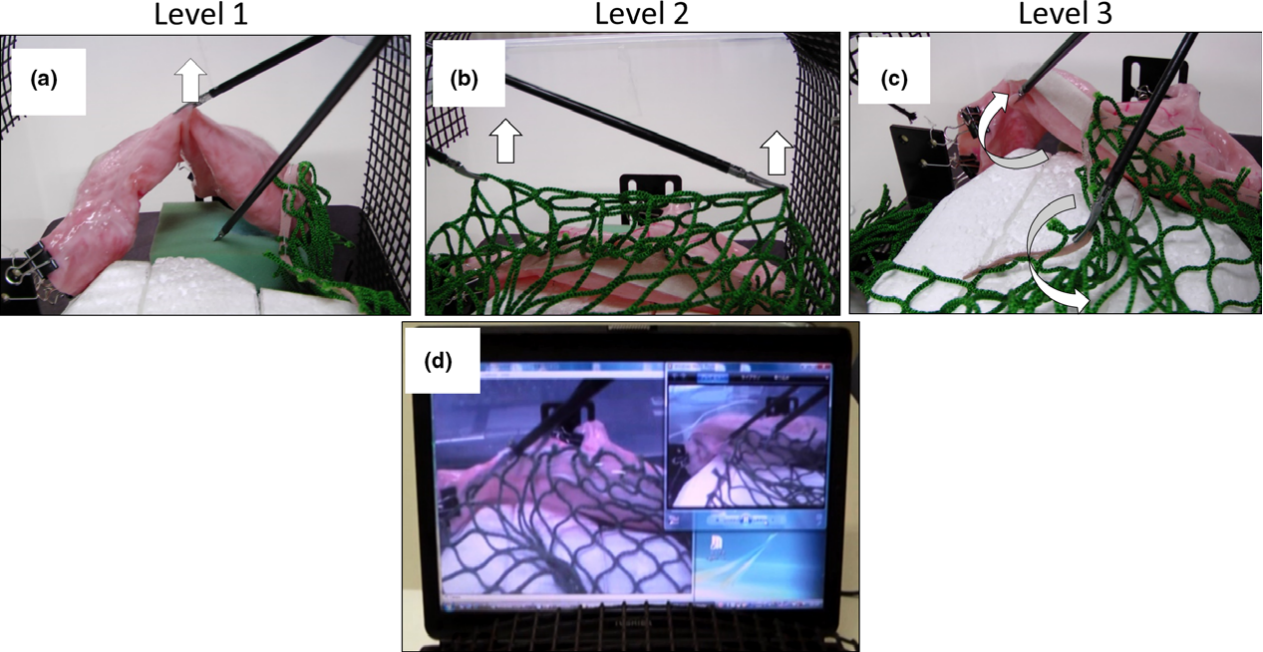
Exemplary demonstrations of the three different tasks. (A) Level 1, flipping over of the stomach from the left side; (B) level 2, simple lifting of the omentum replica using both laparoscopic forceps from the right side; and (C) level 3, lifting and flipping over of the stomach and separation of the omentum from the stomach simultaneously from the left side. (D) The recorded demonstration video displayed on the monitor of the box trainer

Three simple tasks involving organ handling for setting the surgical field were arranged, which simulated the role of a surgical assistant during laparoscopic gastrectomy. The level 1 task involved turning over the stomach from the left side (Figure 2A). The level 2 task involved simple lifting of the omentum replica from the right side using both laparoscopic forceps (Figure 2B). The level 3 task involved lifting and turning over the stomach and separating the omentum from the stomach simultaneously from the left side (Figure 2C). The operator was required to carry out only a lifting motion in the levels 1 and 2 tasks. However, the level 3 task required more complex manipulations such as lifting, flipping, and separating motions. We recorded a video of the precise procedure for each task carried out by a skilled surgeon as an exemplary demonstration. The recorded video was displayed on the monitor of the box trainer (Figure 2D).

### 2.3 Participants

**Figure 3:**
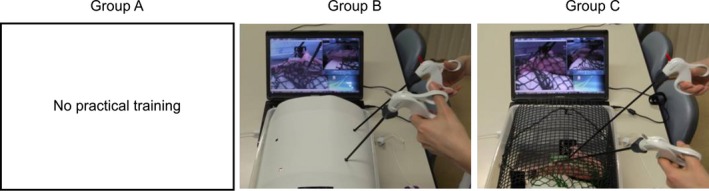
Group A: no practical training; group B: trained using the covered box; and group C: trained using a transparent top that allows for visualization of inside the box

Forty‐five fourth‐year medical students with no previous laparoscopic experience were enrolled in this study. They were randomly divided into three groups with 15 students in each group (Figure 3). Group A, as the untrained group, included students without any practical training before the test. The students of this group were not allowed to carry out any practical training except watching the demonstration videos during the training time. Group B included students with practical training using the box trainer; however, the canopy of the box was covered to conceal the inside. Group C consisted of students with practical training using the transparent box trainer that allows for visualization of the inside of the box during the training. Each of the students in group B or group C carried out practical training using the assigned box trainer. Mean age and sex ratio were equal in the three groups.

Written consent was obtained from the medical students. Ethical approval was obtained from the ethics board/review committee of Kagoshima University.

### 2.4 Assessment

**Figure 4:**
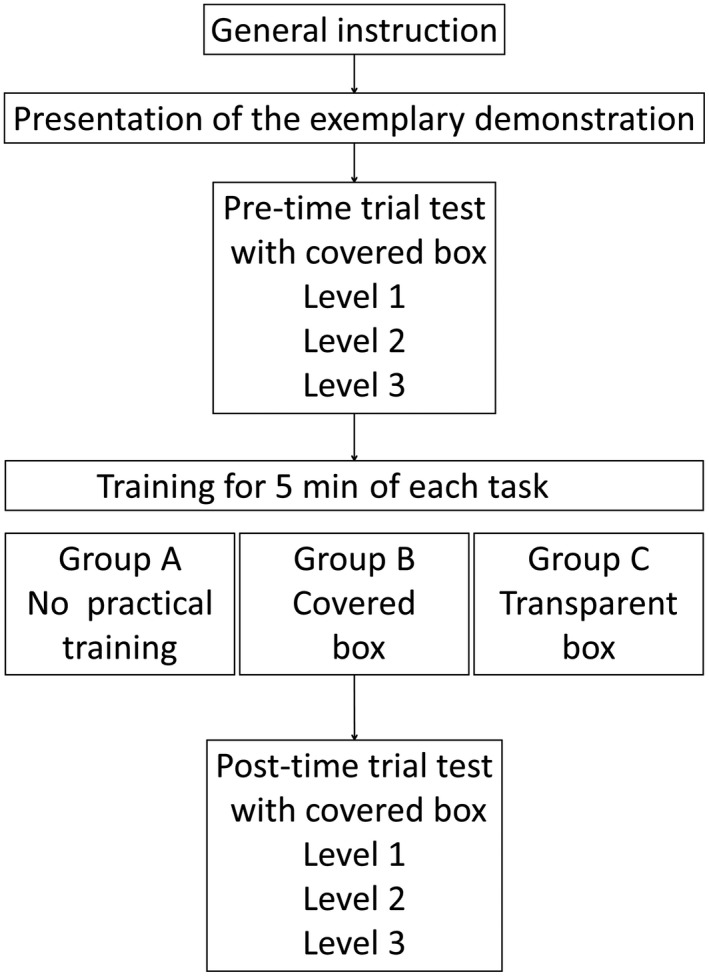
Schematic diagram of the study showing the protocol for assessment and training for each task

Sequence of the assessment protocol is summarized in Figure 4. After participants were informed of the experimental outline, all participants watched the recorded video of the model demonstration three times before the pre‐training time trial test. We carried out all time trial tests using a covered box trainer to simulate the actual operation. All participants carried out each task from levels 1 to 3 as the pre‐training time trial test. Time required to complete each task was measured three times, and the mean value of the three measurements was recorded. Then, each group carried out training time according to each protocol for 5 minutes. Participants, except for those in group A, practiced by themselves to carry out each task while watching the corresponding exemplary demonstration video during the training. After the training, all participants carried out the same tasks again during the post‐training time trial test. Time taken was measured using the same method in each group.

### 2.5 Statistical analysis

We compared the time required to accomplish each task before the training with the time required after the training. The time required to complete each task was calculated as the reduction rate (the post‐test time divided by the pre‐test time) in each group and each task. These data are reported as the mean and standard error. The reduction rate was compared between the no‐training and training groups, and the difference between the groups was calculated using Student's *t* test. Multiple comparisons of the fractional reduction between the three groups were calculated using the Fisher protected least significant difference test. A *P*<.05 was considered statistically significant. Computer software (SPSS, Chicago, IL, USA) was used for all statistical analyses.

## 3 RESULTS

**Figure 5:**
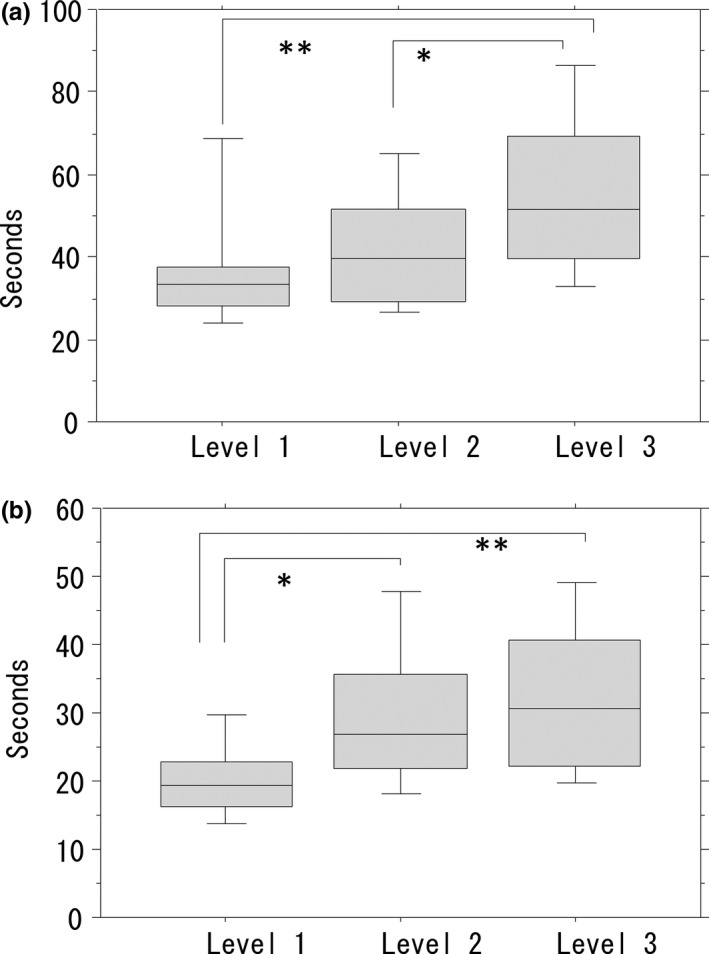
Time required to accomplish each task before and after the training. (A) Level 3 task required significantly more time for operators to complete than did the level 1 (**P<.001) and level 2 tasks (*P<.05) before training. (B) A significant difference in operating time can be observed between the level 1 task and the levels 2 (*P<.001) and 3 tasks (**P<.0001), but not between the levels 2 and 3 tasks after the training

Before the training, the level 3 task required significantly more time to complete than did the levels 1 (*P*<.001) and 2 tasks (*P*<.05) (Figure 5A). Operating time for each task before the training was reduced significantly after the training. There was a significant difference in operating time between the level 1 task and the levels 2 (*P*<.001) and 3 tasks (*P*<.0001), but not between the levels 2 and 3 tasks (Figure 5B).

**Figure 6:**
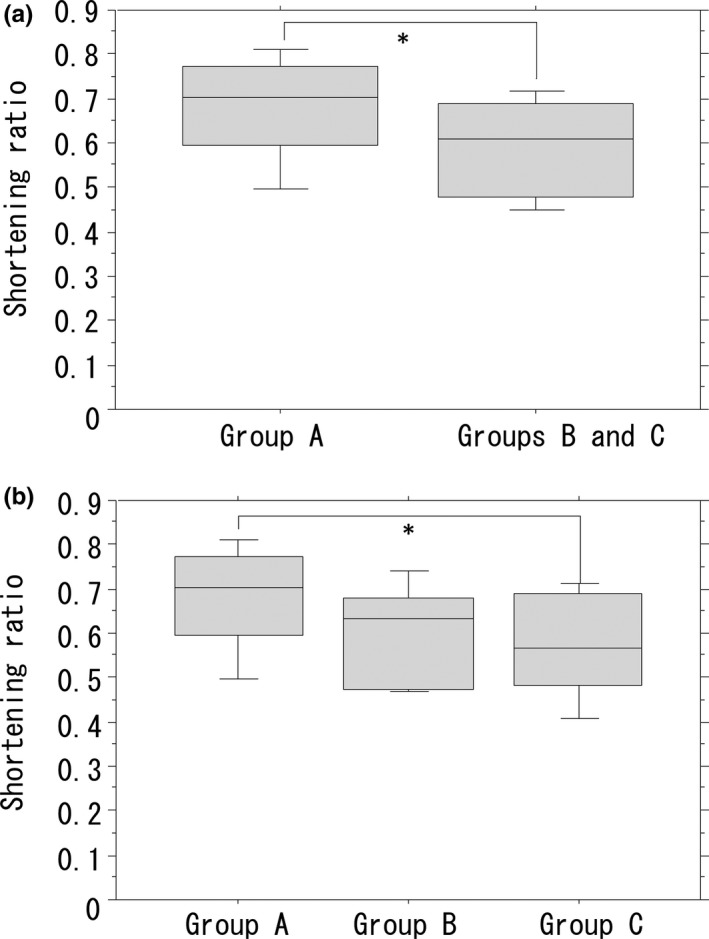
Reduction rates for all groups. (A) Reduction rates in groups B and C are significantly lower than those in group A (*P<.05). Reduction rate in group C was significantly lower than that in group A (*P<.05), but not significantly lower than that in group B

We compared the time reduction rate between the groups. Mean overall time reduction rate was 0.63 (data not shown). Mean reduction rates in groups B and C were significantly reduced as compared with that in group A (Figure 6A). The reduction rate in group C was significantly lower than that in group A; however, the reduction rate in in group B was not significantly lower (Figure 6B).

**Figure 7:**
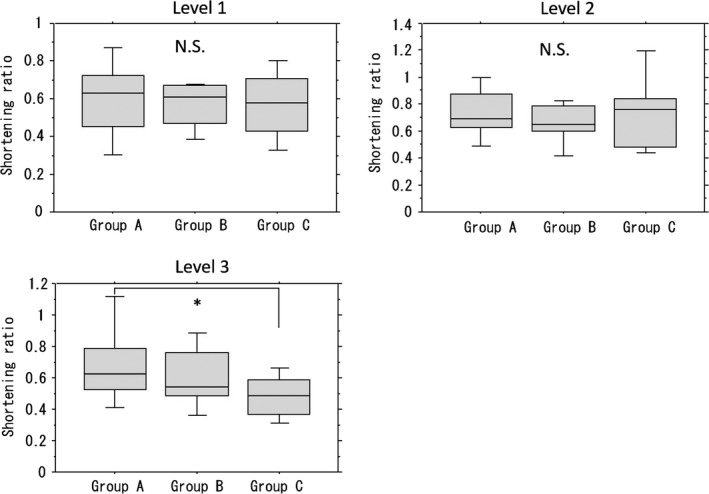
Educational effect of each group on each task. There was no statistically significant difference in the time reduction rate for the levels 1 and 2 tasks between the groups. The reduction rate for the level 3 in group C was lowest, which was statistically significant (*P<.001). N.S., not significant

As described previously, each task had a different level of technical difficulty and so we compared the educational effect of each group in each task. Reduction rates for the level 1 task in groups B and C were slightly lower than the rate in group A, but the difference was not statistically significant. Among the three groups, group B had the lowest time reduction rate, but the difference was not statistically significant. The reduction rate for the level 3 task was lowest in group C, with a statistically significant difference from that in group A (*P*<.001) (Figure 7).

## 4 DISCUSSION

Surgical simulation is now a necessary component of surgical education and is also essential in training surgical residents. Various types of training equipment have been developed to now include features ranging from the simplest box trainer to the expensive computer simulator. However, whether observation over the surgical field during laparoscopic training of novice surgeons is similar to that in open surgery has not yet been investigated. We designed this study to clarify the primitive effect of our original box trainer equipped with a transparent top. Our findings indicate that it is more useful for novices to look over the surgical working field as a visual feedback using a box trainer with a transparent top while carrying out laparoscopic training. The results show that this condition is more effective for mastering a more complex laparoscopic motion. These findings confirm that, during basic training for the laparoscopic procedure, it is preferable for novices as surgical assistants to use a box trainer equipped with a transparent top that enables visualization of the surgical field.

We investigated how visualizing the surgical field affected naive users during training for carrying out laparoscopic operations. Various methods were introduced to objectively evaluate basic laparoscopic surgical skills, such as the virtual reality simulator system and physical trial program.^17^, ^18^, ^19^, ^20^ However, the influence of real‐time visual feedback for laparoscopic surgical training has not yet been reported. We developed the original box trainer, which enables users to see the inside of the box through the completely transparent canopy, to evaluate the effect of visualizing the surgical working field during the operation. We compared the different conditions by switching to the transparent or blinded top of the box trainer using the removable covering canopy.

To proceed smoothly with laparoscopic surgery, the surgical assistant is required to keep the surgical field adequately similar to that during open surgery.^21^, ^22^, ^23^ We considered the training program for surgical assistants as useful for evaluating the surgical training effect in novices. Three types of basic task that require different skills to carry out laparoscopic motions were established in this study. These procedures were thought to be essential motions for assisting operators by providing an adequate surgical field during laparoscopic gastrectomy. We pre‐recorded the exemplary demonstration video of each task for trainees to learn the basic laparoscopic motion. They practiced by imitating the procedure in the demonstration video. Our results indicated that when the procedure became more complex with the addition of even a simple motion, the time for completion of the task was lengthened. We considered this system not only simple for examinations, but also feasible to objectively perceive the degree of upgrade in the skill of laparoscopic surgical assistants for naive users.

Korndorffer *et al*.^24^ evaluated the efficacy of laparoscopic skills training using inexpensive trainer boxes. In this study, operating time after training was significantly reduced not only in the trained groups but also in the untrained group. Even though the untrained students experienced the laparoscopic surroundings only once, most of them became familiar with handling the instruments under video imaging. Interestingly, the time required for completion of the task shortened significantly more quickly in the group that observed the surgical field through a transparent top than in the untrained group, but this was not observed in the trained group that was not allowed to observe the surgical field. This means that trainees are able to complete the task more efficiently while observing the surgical field during laparoscopic surgery. However, in present study, this effect was not demonstrated in the easy task using a simple motion, such as the levels 1 and 2 tasks. We speculate that three‐dimensional (3‐D) visual information of the working field through the transparent top helped to modify the motion in the monitor. A previous report indicated that the mirror visual feedback improved the motor performance of the hand in a neurophysiological study.^25^ Rodrigues *et al*.^19^ reported that novices benefit from starting their training with difficult basic laparoscopic skills in a transparent box trainer. We speculated that one of the important benefits of training using a transparent box may be the visual fusion effect of 3‐D overlooking imaging and conventional two‐dimensional (2‐D) camera imaging. In many reports, novice surgeons were found to perform better and feel more comfortable while carrying out 3‐D laparoscopy than with 2‐D laparoscopy.^26^, ^27^ This result indicates that the real‐time visual feedback enhances the ability of surgeons to perform the laparoscopic technique.

In the present study, we used student volunteers as naïve trainees to clarify the primitive effect of this system. However, there are considerable differences in the amount of clinical work done by students and novice surgeons. Therefore, the result of the experiment may change. An alternative protocol should be investigated using actual novice surgeons to validate the practical effects of the system described herein on laparoscopic training. Moreover, we need to clarify whether our box trainer is useful for the training of more senior surgeons for more complicated procedures, such as laparoscopic gastrointestinal anastomosis and choledochojejunostomy.

The present study has several limitations related to the method used. Although we considered that difficult procedures, such as suturing, were not suitable for naïve trainees in this study, we think that the suitability of the procedure for estimating the laparoscopic skills of surgical assistants should be evaluated. The validation bias depended on the subjective assessment of the judge, particularly in the evaluation of task completion. A digital analysis might be favorable for objectively evaluating the improvement in the performance of the operative procedure using an optical sensor, such as a motion‐tracking system.^20^, ^28^

## 5 CONCLUSIONS

This study shows, for the first time, the significance of using a laparoscopic box trainer with a transparent top to visualize the surgical field during laparoscopic surgical training of naive trainees. The visual feedback on the surgical working field helps novice surgeons become familiar with complex laparoscopic motions. In the future, a randomized trial that is focused on advanced laparoscopic training for experienced surgeons should be conducted with a larger number of subjects.

## DISCLOSURE

Funding: This work was supported by Grants‐in‐aid for Scientific Research (C) [grant number 24501139].

Conflict of Interest: Authors declare no conflicts of interest for this article.

Author Contribution: Kosei Maemura and Shoji Natsugoe designed the study. Kosei Maemura, Yuko Mataki, and Shinichirou Mori developed the methodology. Hiroshi Kurahara, Satoshi Iino, and Masahiko Sakoda collected the data. Kosei Maemura and Hiroyuki Shinchi carried out the analysis. Kosei Maemura wrote the manuscript.
